# Chronic Heart Failure Care Network in Italy: Results from the Regioni 3.0 and ANMCO Heart Failure Working Group Surveys

**DOI:** 10.3390/jcm15218551

**Published:** 2026-11-03

**Authors:** Fabiana Lucà, Alessandro Navazio, Samuela Carigi, Mauro Gori, Giovanna Geraci, Luisa De Gennaro, Concetta Di Nora, Paolo Manca, Maria Vittoria Matassini, Vittoria Rizzello, Maria Denitza Tinti, Matteo Bianco, Renata De Maria, Vittorio Palmieri, Daniela Aschieri, Ilaria Battistoni, Claudio Bilato, Stefania Angela Di Fusco, Vered Gil Ad, Attilio Iacovoni, Barbara Maria Teresa Mabritto, Massimo Milli, Pietro Scicchitano, Francesco Vigorito, Michele Massimo Gulizia, Furio Colivicchi, Fabrizio Oliva, Federico Nardi, Massimo Grimaldi

**Affiliations:** 1 U.O.C. Cardiologia-UTIC, Grande Ospedale Metropolitano GOM, 890129 Reggio Calabria, Italy; 2 S.O.C. Cardiologia Ospedaliera, Presidio Ospedaliero Arcispedale Santa Maria Nuova, Azienda USL di Reggio Emilia—IRCCS, 42123 Reggio Emilia, Italy; alessandro.navazio@ausl.re.it; 3 U.O. Cardiologia, Ospedale Infermi Rimini, AUSL della Romagna, 47923 Rimini, Italy; samuela.carigi@gmail.com; 4 U.O.C. Cardiologia 1, Dipartimento Cardiovascolare, ASST Papa Giovanni XXIII, 24127 Bergamo, Italy; 5 U.O. Cardiologia, P.O. Sant’Antonio Abate, ASP Trapani, 91016 Erice, Italy; 6 U.O.C. Cardiologia, Ospedale San Paolo, 70132 Bari, Italy; luisadegennaro@hotmail.com; 7 S.O.C. Cardiochirurgia, Azienda Sanitaria Universitaria Friuli Centrale, 33100 Udine, Italy; concetta.dinora@asufc.sanita.fvg.it; 8 Cardiologia, Istituto Mediterraneo per i Trapianti e Terapie ad Alta Specializzazione, ISMETT IRCCS, 90127 Palermo, Italy; 9 S.O.D. Cardiologia-UTIC, A.O.U. delle Marche, 60126 Ancona, Italy; mariavittoria.matassini@ospedaliriuniti.marche.it (M.V.M.);; 10 U.O.C. Cardiologia, A.O. San Giovanni Addolorata, 00184 Roma, Italy; 11 U.O.C. Cardiologia, A.O. San Camillo Forlanini, 00152 Roma, Italy; denitzatinti@gmail.com; 12 S.C.D.O. Cardiologia, A.O.U. San Luigi Gonzaga, 10043 Orbassano, Italy; m.bianco@sanluigi.piemonte.it; 13 Dipartimento Cure Primarie ASST Fatebenefratelli Sacco, 20157 Milano, Italy; renata_de_maria@hotmail.com; 14 U.O.C. Cardiochirurgia, A.O. S. Anna e S. Sebastiano, 81100 Caserta, Italy; 15 U.O.C. Cardiologia e UTIC, Ospedale “Guglielmo da Saliceto”, 29121 Piacenza, Italy; dani.aschieri@gmail.com; 16 U.O.C. Cardiologia, Ospedali dell’Ovest Vicentino, Azienda ULSS 8 Berica, 36100 Vicenza, Italy; claudio.bilato@aulss8.veneto.it; 17 U.O.C. Cardiologia Clinica e Riabilitativa, Presidio Ospedaliero San Filippo Neri— ASL Roma 1, 00135 Roma, Italy; stefania.difusco@aslroma1.it (S.A.D.F.); furio.colivicchi@gmail.com (F.C.); 18 IRCCS Ospedale Policlinico San Martino di Genova, Department of Cardio-Thoraco-Vascular Diseases—Cardiology Area, Largo Rosanna Benzi 10, 16132 Genova, Italy; 19 Department of Transplant Surgery and Surgical Treatment of Heart Failure, Azienda Socio Sanitaria Territoriale Papa Giovanni XXIII, 24127 Bergamo, Italy; 20 Cardiologia, Azienda Ospedaliera Ordine Mauriziano, 10128 Torino, Italy; barbara.mabritto@gmail.com; 21 Cardiologia Firenze 1 (Ospedali S. Maria Nuova e Nuovo San Giovanni di Dio), Azienda USL Toscana Centro, 50100 Florence, Italy; massimo.milli@uslcentro.toscana.it; 22 Divisione di Cardiologia, Ospedale “F. Perinei” ASL BA, 70022 Altamura, Italy; pietro.scicchitano@asl.bari.it; 23 Divisione di Cardiologia, S. Giovanni Di Dio E Ruggi D’Aragona Hospital of Salerno, Largo Città di Ippocrate, 84131 Salerno, Italy; francesco.vigorito@icloud.com; 24 U.O.C. Cardiologia, Ospedale Garibaldi-Nesima, Azienda di Rilievo Nazionale e Alta Specializzazione “Garibaldi”, 95122 Catania, Italy; michele.gulizia60@gmail.com; 25 Cardiologia 1-Emodinamica, Dipartimento Cardiotoracovascolare “A. De Gasperis”, ASST Grande Ospedale Metropolitano Niguarda, 20162 Milano, Italy; fabrizio.oliva@ospedaleniguarda.it; 26 Dipartimento di Cardiologia, Ospedale Santo Spirito, Casale Monferrato, 15033 Alessandria, Italy; federico.nardi1@gmail.com; 27 Ente Ecclesiastico Ospedale Generale Regionale “ F. Miulli”, 70021 Acquaviva delle Fonti, Italy; m.grimaldi@miulli.it

**Keywords:** chronic heart failure, care network, hospital–territory connections, follow-up, multidisciplinary approach

## Abstract

**Background:** Heart failure (HF) remains a major cause of morbidity, mortality, and healthcare resource utilisation, requiring integrated multidisciplinary care across hospital and community settings. This study aimed to describe the organisation of HF care and post-discharge management in Italy through two complementary national surveys promoted by the Italian Association of Hospital Cardiologists (ANMCO). **Methods:** Two complementary cross-sectional organisational surveys were conducted to describe the organisation of HF care in Italy. The Regioni 3.0 survey, performed between November 2023 and September 2024, collected 196 responses from directors of cardiology departments and HF Centers regarding organisational resources, post-discharge follow-up pathways, and integration with community healthcare services. A second survey, administered during the ANMCO Heart Failure Centers Convention in October 2024, collected 135 responses on discharge planning and outpatient follow-up practices. **Results:** HF outpatient clinics were available in 85.7% of Centers, whereas only 42.6% reported non-physician staff specifically assigned to HF care. Structured post-discharge follow-up pathways were reported by 91.3% of Centers, but functional links with primary care or community healthcare services were present in only 47.7%. Medication reconciliation and patient education were routinely performed in most Centers, whereas telemonitoring systems were available in approximately one-third of respondents. **Conclusions:** HF care in Italy is supported by a widespread network of outpatient clinics and structured post-discharge follow-up pathways. However, important organizational gaps remain in multidisciplinary staffing, hospital–community integration, and telemonitoring, highlighting the need to strengthen multidisciplinary resources, improve hospital–community integration, and expand telemonitoring to support more coordinated HF care nationwide.

## 1. Introduction

Heart failure (HF) represents one of the leading causes of mortality, morbidity, and healthcare costs in industrialised countries [1,2,3,4,5].

In Italy, HF is the third leading cause of hospitalisation in the general population and the leading cause among individuals older than 65 years. HF accounts for more than three-quarters of total healthcare expenditure related to cardiovascular diseases (CVD) [6].

The natural history of HF is characterised by periods of relative clinical stability interspersed with episodes of acute decompensation. Outcomes are substantially worse following a hospitalisation for acute HF (HFH), with mortality rates of approximately 20–25% during the first year after discharge [7,8] and readmission rates estimated at 25% at 30 days and 36% at 90 days [8].

According to the most recent report of the National Outcomes Program (Programma Nazionale Esiti, PNE), approximately 155,000 hospital admissions for HF occur annually in Italy, of which more than 116,000 are related to acute HF. Thirty-day mortality after hospitalisation is reported at 9.4%, while the 30-day readmission rate is 11.9%. Although these indicators have progressively improved compared with 2015 (mortality approximately 11%; readmissions approximately 14%), substantial regional variability persists. These findings confirm the considerable clinical and organisational burden of HF within the Italian healthcare system and highlight the need for structured and integrated post-discharge care pathways [9].

Patients with HF commonly present with multiple cardiovascular (CV) and non-CV comorbidities [10,11]. According to the recommendations of the Italian National Institute of Health (Istituto Superiore di Sanità, ISS), HF management should follow a multidisciplinary approach involving cardiologists, nurses, internists, geriatricians, general practitioners (GPs), rehabilitation specialists, palliative care physicians, psychologists, as well as patients’ family members and caregivers [7,12,13,14,15].

Outpatient follow-up plays a crucial role in preventing readmissions and improving clinical outcomes. In particular, the discharge phase represents a key opportunity to implement shared care pathways [16], enabling optimisation and up-titration of guideline-directed medical therapy, promotion of patient self-management skills, and planning of further diagnostic assessments [17,18].

Within this framework, dedicated HF networks may play a central role [19,20] in bridging hospital and community settings, facilitating continuity of care, improving treatment adherence, and reducing the risk of rehospitalisation. Building on a national survey providing a snapshot of current organisational models, the present study aimed to describe the organisation of HF care in Italy, identify potential gaps in hospital–community integration, and support the development of more coordinated and multidisciplinary care pathways [8,21].

## 2. Methods

This study was based on two complementary national surveys promoted by Italian Association of Hospital Cardiologists (ANMCO) and aimed at describing the organisation of HF care and post-discharge management practices in Italy.

The first survey was conducted within the framework of the Regioni 3.0 project and focused on the organisational characteristics of HF services, including outpatient clinics, dedicated personnel, post-discharge follow-up pathways, and integration with community healthcare services. The second survey, promoted by the ANMCO Heart Failure Working Group, explored selected aspects of discharge planning and outpatient follow-up management in patients hospitalised for acute HF.

From November 2023 to September 2024, ANMCO promoted the Regioni 3.0 project, a nationwide initiative designed to map and compare organisational models of CV care across Italian cardiology departments. The project aimed to identify potential disparities in healthcare delivery, promote shared care pathways, and facilitate the progressive alignment of organisational models according to homogeneous quality standards throughout the national territory.

Within the Regioni 3.0 project, the HF survey consisted of 14 items focused on the outpatient management of patients with HF. The questionnaire was addressed to Directors of Complex Cardiology Units, Heads of HF Centers, and other cardiologists with direct organisational responsibilities who were ANMCO members and distributed across the Regional Sections (Supplementary Table S1). The complete questionnaire is provided as Supplementary Table S1 to facilitate the interpretation and reproducibility of the survey.

No restriction was imposed to limit responses to a single entry per center; therefore, more than one response could originate from the same institution. This methodological choice was intended to maximise participation and capture organisational perspectives from individuals directly involved in HF care. Given the organisational nature of the survey, responses originating from the same institution were expected to describe the same organisational setting.

Unless otherwise specified, survey items allowed a single response. Questions regarding the presence of structured post-discharge follow-up pathways (item 10) and functional links with community healthcare services (item 7) were formulated as dichotomous (Yes/No) variables and referred to the organisational characteristics of the responding institution. Conversely, for the item concerning referral pathways (item 9), respondents were allowed to select multiple answers because patients may access HF services through different routes; therefore, percentages for this item are not mutually exclusive and were calculated using the total number of respondents as the denominator. No formal verification of organisational structures, institutional resolutions, or written protocols was performed. Overall, the survey was distributed to 563 ANMCO members with organisational responsibilities in HF care. A total of 196 responses were collected, corresponding to a response rate of 34.8%.

The geographical distribution of respondents reflected the national distribution of ANMCO cardiology facilities, with a higher concentration in Northern Italy, consistent with the greater density of cardiology centers in those regions.

The second survey, promoted by the ANMCO Heart Failure Working Group, was administered during the Heart Failure Centers Convention held in Milan on 25–26 October 2024. This survey consisted of six single-response, closed-ended items exploring selected aspects of discharge planning and outpatient follow-up management in patients hospitalised for acute HF: routine medication reconciliation at discharge (item 1); availability of a dedicated outpatient HF clinic (item 2); availability of a dedicated HF nurse (item 3); proportion of patients reassessed in an outpatient setting within one month of discharge (item 4); systematic assessment of therapeutic adherence and/or provision of educational interventions on therapy and self-monitoring (item 5); and availability of a telemonitoring system during the post-discharge vulnerable period (item 6),the latter adopting a deliberately broad operational definition encompassing heterogeneous modalities (e.g., televisit, structured telephone call, other) (Supplementary Table S2). The complete questionnaire is provided as Supplementary Table S2 to facilitate interpretation and reproducibility. For items 4 and 5, respondents without an outpatient HF clinic could select a ‘not applicable’ option; these responses were excluded from the denominator when calculating item-specific percentages, and this is indicated in the corresponding footnote.

The two surveys were conceived as complementary but methodologically distinct tools, with different modes of administration and potentially different respondent populations. Individual participant tracking was not performed; therefore, respondent overlap between the two surveys could not be assessed and direct comparisons between datasets were not undertaken.

To facilitate interpretation of the findings, responses from the Regioni 3.0 survey were stratified according to macro-geographical working group (North, Center, and South & Islands) and center type (cardiology units with inpatient beds and intensive cardiac care unit (ICCU); cardiology units with inpatient beds without ICCU; cardiology units without inpatient beds; and other organisational settings).

Categorical variables were summarised as absolute frequencies and percentages. Comparisons between groups were performed using the χ^2^ test and should be considered exploratory given the voluntary nature of participation and the possibility that more than one response may originate from the same institution. For selected variables, post-hoc pairwise comparisons between geographical macro-areas were performed using the χ^2^ test, applying a Bonferroni correction for multiple comparisons (adjusted significance threshold *p* < 0.017). Because the questionnaire was administered anonymously and institutional identifiers were not collected, formal deduplication at the center level was not feasible. As a proxy, a post-hoc sensitivity analysis was performed, in which potential duplicate responses were conservatively defined as responses originating from the same region and reporting identical answers to all 14 survey items; the main organisational estimates were then recalculated after their exclusion. The association between monthly HF patient volume and the availability of a dedicated HF outpatient clinic or of dedicated non-physician staff, including dedicated HF nurses, was assessed using the χ^2^ test. A two-sided *p*-value < 0.05 was considered statistically significant. Percentages were calculated using the number of available responses for each item as the denominator, with missing data accounted for where applicable. For item 11 of the Regioni 3.0 survey, which was conditional on reporting a structured post-discharge follow-up pathway (item 10), five respondents answered despite reporting no structured pathway; these responses were excluded, and percentages were calculated among the 179 respondents with a structured follow-up pathway

## 3. Results

The ANMCO Regioni 3.0 survey collected 196 responses from cardiologists with organisational responsibilities in HF care across Italy. The characteristics of respondents and participating centers are summarised in Table 1.

**Figure 1:**
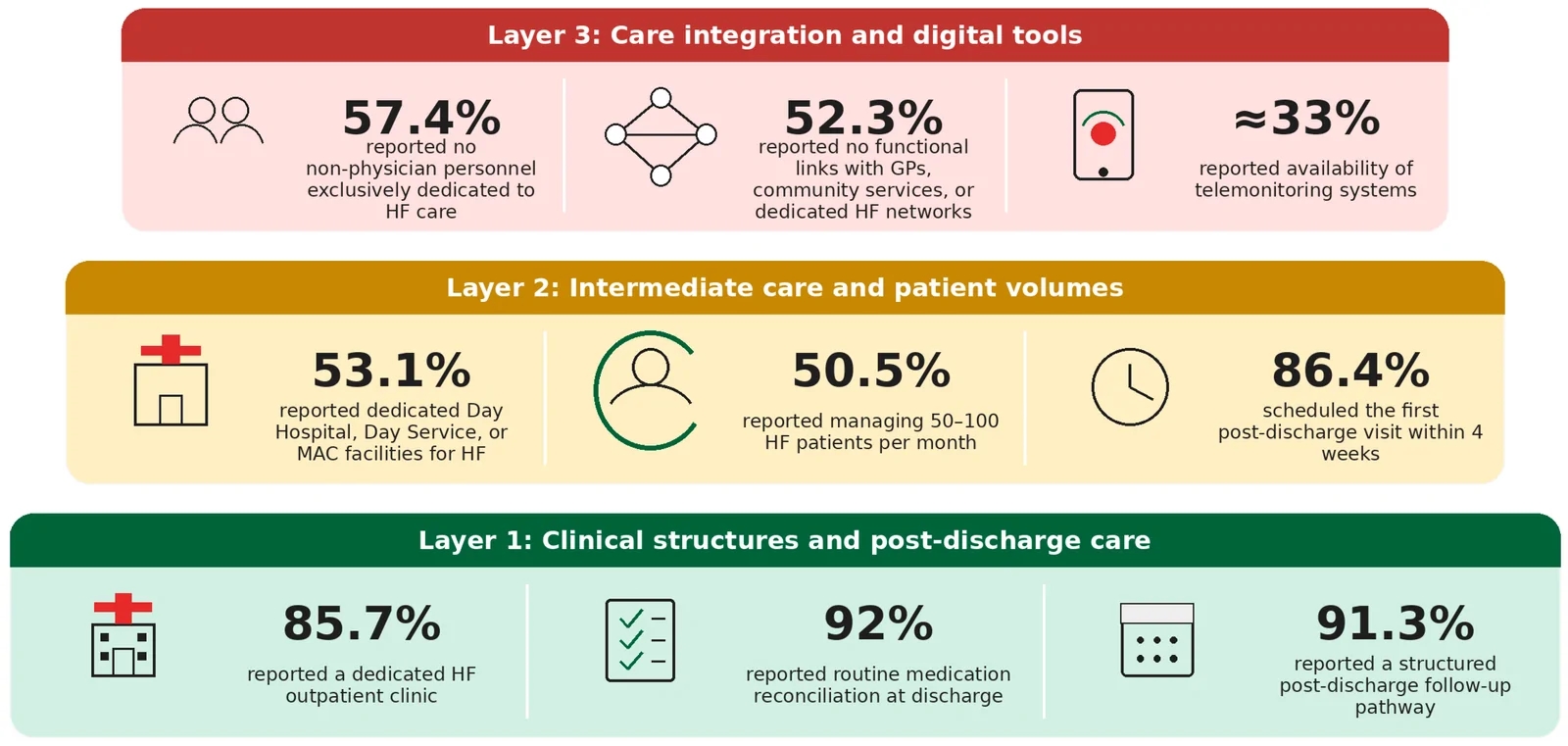
Organisation of HF care in Italy: from clinical structures to care integration.

Abbreviations: HF: heart failure; GP: general practitioner; MAC: complex outpatient activity unit.

To explore potential geographical disparities in HF care organisation, the main organisational variables were stratified according to macro-geographical working group (North, Center, and South & Islands) (Table 2). The overall organisation of HF care emerging from the survey is summarised in Figure 1.

The figure summarises selected organisational findings from the Regioni 3.0 and ANMCO Heart Failure Working group surveys. Dedicated HF outpatient clinics, medication reconciliation at discharge, and structured post-discharge follow-up pathways were frequently reported, indicating that core components of HF care are already present in many Italian cardiology centers. By contrast, dedicated non-physician personnel, functional links with GPs or community healthcare services, and telemonitoring systems were less consistently available. These findings suggest that further improvement should focus on strengthening multidisciplinary resources and improving integration between hospital-based HF services and community care.

Dedicated HF outpatient clinics were highly prevalent throughout the country, being reported by 89.8% of centers in Northern Italy, 87.5% in Central Italy, and 77.6% in Southern Italy and the Islands, without statistically significant differences (*p* = 0.102). Similarly, the availability of dedicated HF nurses did not differ significantly across geographical areas (45.4%, 25.0%, and 36.2%, respectively; *p* = 0.077). In post-hoc pairwise comparisons, the availability of dedicated HF nurses was nominally higher in Northern than in Central Italy (45.4% vs. 25.0%; unadjusted *p* = 0.027); however, this difference did not remain statistically significant after Bonferroni correction for multiple comparisons (adjusted significance threshold *p* < 0.017). No significant differences emerged between Northern and Southern Italy & Islands (*p* = 0.264) or between Central and Southern Italy & Islands (*p* = 0.241).

Conversely, significant geographical differences emerged in the availability of Day Hospital/Day Service or complex outpatient activity unit (MAC) facilities dedicated to HF patients (64.3%, 40.0%, and 43.1%, respectively; *p* = 0.007) and in the presence of functional links with GPs, community healthcare services, or dedicated care networks (61.9%, 27.5%, and 37.9%, respectively; *p* < 0.001), both of which were more frequently reported in Northern Italy (Table 2). These geographical patterns are summarised in Supplementary Figure S1. Stratification according to center type demonstrated that dedicated HF outpatient clinics were more frequently available in cardiology departments with inpatient beds and ICCU (91.3%) compared with cardiology departments with inpatient beds but without ICCU (75.0%), cardiology units without inpatient beds (60.0%), and other organisational settings (53.8%) (*p* < 0.001). Similarly, structured post-discharge follow-up pathways were more frequently reported in centers with ICCU (96.6%) than in the other organisational settings (*p* < 0.001). No significant differences were observed across center types regarding the availability of dedicated non-physician staff, dedicated nurses, Day Hospital/MAC facilities, or functional links with community healthcare services (Table 3).

Overall organisational characteristics are reported in Table 1. Among respondents, 85.7% reported having a dedicated HF outpatient clinic in their institution, while non-physician professionals dedicated exclusively to HF care were available in 42.6% of centers, primarily nurses (38.5%). Day Hospital/Day Service or MAC facilities dedicated to HF patients were available in 53.1% of centers, whereas functional links with GPs, community healthcare services, or dedicated networks were reported by 47.7% of respondents.

Regarding patient volume, 39.3% of respondents reported managing fewer than 50 HF patients per month, 50.5% reported between 50 and 100 patients per month, and 10.2% reported more than 100 patients per month (Table 1). The presence of a dedicated HF outpatient clinic was significantly associated with monthly HF patient volume, being reported by 67.5% (52/77) of centers managing fewer than 50 patients per month, 99.0% (98/99) of centers managing 50–100 patients, and 90.0% (18/20) of centers managing more than 100 patients per month (*p* < 0.001). Similarly, the availability of non-physician professionals dedicated to HF care increased with patient volume, from 26.3% (20/76) in centers managing fewer than 50 patients per month to 50.5% (50/99) and 65.0% (13/20) in centers managing 50–100 and more than 100 patients per month, respectively (*p* < 0.001). Likewise, the availability of dedicated HF nurses increased significantly with monthly patient volume, from 21.1% (16/76) in centers managing fewer than 50 patients per month to 46.5% (46/99) and 65.0% (13/20) in centers managing 50–100 and more than 100 patients per month, respectively (*p* < 0.001). In the post-hoc sensitivity analysis, only 4 potential duplicate responses (2.0%) were identified. After their exclusion (*n* = 192), the main organisational estimates remained essentially unchanged (dedicated HF outpatient clinic: 85.7% vs. 85.4%; structured post-discharge follow-up: 91.3% vs. 91.1%; functional links with community services: 47.7% vs. 47.6%; all differences ≤0.3 percentage points), suggesting that potential institutional duplication is unlikely to have materially affected the results.

Referral pathways and post-discharge follow-up organisation are summarised in Table 4. HF patients were referred to participating centers through multiple pathways, including the emergency department (69.4%), GP outpatient clinics (50.0%), internal medicine or geriatrics departments (49.5%), territorial cardiology clinics (42.9%), and other sources (37.2%); as multiple responses were allowed for this item (item 9), percentages are not mutually exclusive.

Overall, 91.3% of respondents reported the availability of a structured post-discharge follow-up pathway. The first post-discharge visit was scheduled within 4 weeks in 88.3% of cases (158/179). Regarding the timing of reassessment after discharge, respondents reported follow-up after 1 week in 7.8% of cases, after 1 month in 78.2%, after 6 months in 8.8%, and after 12 months in one case (0.5%). In 4.7% of cases, patients were referred to another center, a GP, or a private cardiologist rather than being followed by the same institution.

For follow-up management, 85.6% of respondents reported referral back to the HF outpatient clinic of the same hospital, 5.7% to a community cardiologist, 4.6% to a GP, 2.6% to a private cardiologist, and 1.5% to private or intramural outpatient services. During the year following discharge, respondents reported reassessing patients on average three times in 49.5% of cases, twice in 32.5%, four or more times in 11.3%, and once in 5.2%; only 1.5% reported no reassessment (Table 4). Referral pathways and post-discharge follow-up organisation are graphically summarised in Supplementary Figure S2.

To complement the organisational findings of the Regioni 3.0 survey, the ANMCO Heart Failure Working Group conducted a second survey focused on discharge planning and outpatient follow-up practices. A total of 135 responses were collected (Table 5).

Among the 128 (item 4) and 131 (item 5) respondents reporting the availability of a dedicated outpatient HF clinic, respondents who selected ‘not applicable’ were excluded from the denominator.

Overall, 91.9% of respondents reported that medication reconciliation involving the patient and/or caregiver was routinely performed at discharge by a physician and/or nurse. A dedicated outpatient HF clinic was available in 86.7% of centers, and a dedicated HF nurse in 43.7%. Among respondents with an outpatient HF clinic, patients hospitalised for HF were reassessed within one month of discharge in more than 75% of cases at 25.8% of centers, in 50–75% of cases at 21.9%, in 25–50% of cases at 20.3%, and in fewer than 25% of cases at 32.0%. Among the same subgroup, therapeutic adherence and/or educational interventions on treatment and self-monitoring were systematically provided in 87.0% of centers (46.6% both adherence assessment and education; 23.7% adherence assessment only; 16.8% educational intervention only), while 13.0% reported neither. Only 34.1% of respondents reported the availability of a telemonitoring system during the post-discharge vulnerable period.

## 4. Discussion

The two surveys provide complementary information on the current organisation of HF care in Italy, addressing both the structural characteristics of HF services and selected aspects of discharge planning and outpatient follow-up. Although based on different methodologies and involving potentially different respondent populations, the findings of these two complementary exploratory surveys provide a descriptive overview of contemporary HF management in Italian cardiology practice and identify several organisational strengths and areas requiring further development [22].

The Regioni 3.0 survey demonstrates that specialised outpatient HF services are widely available across the country. Dedicated HF outpatient clinics were reported by 85.7% of participating centers, confirming that structured ambulatory management has become a central component of HF care. This finding is consistent with the increasing emphasis placed by international guidelines on longitudinal disease management, optimisation of guideline-directed medical therapy (GDMT), patient education, and continuity of care after hospital discharge [13,14]. The broad availability of dedicated HF clinics observed in the present survey suggests that the organisational framework required to support these activities is already established in most Italian cardiology centers. Notably, the availability of a dedicated outpatient HF clinic (86.7%) and of a dedicated HF nurse (43.7%) reported in the second survey were consistent with the corresponding findings of the Regioni 3.0 survey (85.7% and 38.5%, respectively), despite the two surveys involving different administration modes and largely non-overlapping respondent samples. This concordance across two independent surveys strengthens confidence in the robustness of these organisational estimates.

However, the availability of specialised outpatient services was not paralleled by a similar diffusion of dedicated multidisciplinary resources. Non-physician healthcare professionals specifically assigned to HF care were available in only 42.6% of participating centers, while dedicated HF nurses were reported by 38.5% of respondents. These findings are particularly relevant because HF management increasingly requires coordinated interventions extending beyond pharmacological treatment alone. Previous studies have shown that specialised HF nurses contribute substantially to patient education, reinforcement of treatment adherence, recognition of early signs of clinical deterioration, optimisation of therapeutic pathways, and coordination between healthcare professionals involved in patient care [13,14]. Therefore, the limited availability of dedicated personnel identified by the survey may represent a significant organisational constraint despite the widespread presence of specialised outpatient clinics. Another important component of multidisciplinary HF management is cardiac rehabilitation [23]. Although current guidelines recommend its integration into comprehensive HF care pathways, this aspect was not specifically investigated in the present surveys and therefore deserves dedicated evaluation in future national organisational studies.

The survey also revealed important differences in the availability of intermediate-care facilities. Dedicated Day Hospital, Day Service, or complex outpatient activity units (MAC) for HF patients were available in 53.1% of centers. Consequently, nearly half of participating institutions lacked dedicated organisational structures capable of managing selected episodes of clinical instability without resorting to conventional hospital admission. Such facilities may facilitate treatment optimisation, the administration of intravenous therapies, the reassessment of worsening symptoms, and the management of patients requiring close monitoring but not necessarily inpatient care. Their uneven distribution across the healthcare system may therefore limit the implementation of more flexible care pathways and contribute to differences in local organisational capacity.

Another notable finding concerns the organisation of post-discharge follow-up. Structured follow-up pathways were reported by 91.3% of centers, while 88.3% indicated that the first outpatient reassessment was routinely scheduled within four weeks after discharge. These figures suggest that most participating centers recognise the importance of early post-discharge evaluation, a phase that is associated with an increased risk of adverse events and rehospitalisation [24,25,26,27,28,29]. At the same time, the slight discrepancy between the availability of structured pathways and their reported timing highlights the distinction between organisational planning and actual implementation, which may be influenced by staffing resources, outpatient capacity, local logistics, and patient-related factors.

Perhaps the most important organisational issue emerging from the survey concerns the relationship between hospital-based HF services and community healthcare structures. Functional links with general practitioners (GPs), territorial healthcare services, or dedicated HF networks were reported by only 47.7% of respondents. Consequently, more than half of participating centers declared the absence of formalised collaborative pathways with community-based providers. This finding is particularly relevant because effective HF management requires continuous interaction among different levels of care, especially during transitions between hospital and outpatient settings. As illustrated conceptually in Figure 1, the survey suggests that while the structural components of HF care are largely established, integration between specialised cardiology services and territorial healthcare networks remains less developed. Stratification according to geographical macro-working group provides additional insight into the organisational characteristics of HF services across Italy. Notably, the availability of dedicated HF outpatient clinics was consistently high in Northern, Central, and Southern regions, with no statistically significant differences among macro-areas. This observation suggests that specialised outpatient HF care has become a widely adopted organisational model throughout the country and represents one of the most homogeneous components of HF service provision.

In contrast, significant differences emerged for several organisational variables related to resource allocation and care integration. The availability of dedicated non-physician personnel varied significantly across geographical areas, being more frequently reported in Northern regions than in Central or Southern Italy (*p* = 0.022). A similar trend was observed for dedicated HF nurses, although the difference did not reach statistical significance. Although differences in dedicated personnel were observed, Supplementary Figure S1 focuses on organisational variables that showed the greatest impact on care integration. While these findings should be interpreted cautiously, they suggest that the diffusion of multidisciplinary HF teams may be less uniform than the availability of dedicated outpatient clinics. Given the recognised contribution of specialised personnel to patient education, treatment optimisation, and coordination of care, these differences may have important implications for the delivery of comprehensive HF management programmes [13,14].

Geographical variability was also evident in the availability of intermediate-care facilities. Centers located in Northern Italy more frequently reported the presence of Day Hospital, Day Service, or MAC structures dedicated to HF management. Likewise, formalised links with GPs, community healthcare services, and organised HF networks were significantly more common in Northern regions. These findings suggest that the principal source of geographical heterogeneity is not represented by access to specialised cardiology services themselves, but rather by the organisational infrastructure supporting continuity of care and integration between different healthcare settings.

The analysis according to center type provides additional insight into the factors influencing organisational development. Dedicated HF outpatient clinics were significantly more common among cardiology departments with inpatient beds and ICCU, where specialised HF services were reported by the vast majority of respondents. Similarly, structured post-discharge follow-up pathways were more frequently available in centers with inpatient facilities and ICCU support. These findings are not unexpected and likely reflect differences in organisational complexity, patient volume, staffing resources, and availability of specialised expertise.

In particular, cardiology departments with inpatient beds and an ICCU are typically located in larger hospitals and are staffed by a considerably greater number of cardiologists, allowing dedicated medical time and personnel to be allocated to a structured HF outpatient clinic. Conversely, cardiology units without inpatient beds usually operate in smaller hospitals with a limited number of cardiologists, who are often required to cover the entire spectrum of cardiovascular care; in this setting, the human resources needed to establish and maintain a dedicated HF outpatient clinic may simply not be available. Rather than reflecting an organisational shortcoming of individual centers, this pattern underscores the importance of hub-and-spoke network models, in which smaller units without dedicated HF clinics are functionally linked to larger referral centers, ensuring that patients managed in these settings retain access to structured HF care and post-discharge follow-up.

Interestingly, several organisational limitations appeared to be shared across different institutional settings. The availability of dedicated non-physician personnel, dedicated HF nurses, intermediate-care facilities, and formalised territorial networks remained suboptimal irrespective of center type. This observation suggests that some of the challenges identified by the survey are not exclusively related to the size or complexity of individual institutions but may instead reflect broader structural issues affecting HF care delivery at a national level. In this regard, the analysis according to monthly patient volume provides useful complementary information. The availability of dedicated HF nurses increased progressively with patient volume, suggesting that in smaller, low-volume centers the absence of dedicated figures may largely reflect the limited number of professionals working in the cardiology unit. However, even among high-volume centers, more than one-third lacked a dedicated HF nurse, indicating that the shortage of dedicated personnel cannot be attributed to workforce size alone and also reflects insufficient organisational investment in structured, nurse-based HF care models.

The analysis of referral pathways provides additional insight into the current organisation of HF care in Italy. Patients reached specialised HF services through multiple routes, including emergency departments, GP outpatient clinics, internal medicine or geriatrics departments, territorial cardiology services, and other referral sources. Because multiple responses were allowed, these findings should not be interpreted as mutually exclusive pathways but rather as indicators of the complexity of the current healthcare network. The coexistence of several access routes suggests that entry into specialised HF care often depends on local organisational structures and referral practices rather than on a standardised national pathway.

This heterogeneity is also reflected in the organisation of post-discharge management. The vast majority of respondents reported that patients were referred back to the HF outpatient clinic of the same institution following hospital discharge, whereas referral to community cardiologists, GPs, or private outpatient services was relatively uncommon. These findings indicate that continuity of care is predominantly maintained within hospital-based cardiology services. On one hand, this organisational model may facilitate therapeutic optimisation, ensure access to specialised expertise, and promote consistency in clinical decision-making. On the other hand, it may also reflect the limited development of shared-care models involving territorial healthcare providers. Integration with territorial providers is particularly important for older patients with multiple comorbidities, who frequently require coordinated interventions extending beyond CV care alone [12,13,14,15].

Another noteworthy finding concerns the volume of HF patients managed by participating centers. Approximately half of respondents reported following between 50 and 100 HF patients per month, whereas around 40% reported fewer than 50 patients monthly and only a minority reported volumes exceeding 100 patients. Although these data do not distinguish between incident and prevalent cases and should therefore be interpreted cautiously, they suggest considerable variability in the distribution of HF care activity across the national territory. Such variability may reflect differences in population density, referral patterns, local healthcare organisation, availability of specialist services, and the degree of involvement of non-cardiology providers in patient management.

The organisational implications of these findings may be considered in light of previous evidence regarding specialised HF care. Lindberg et al. demonstrated that a substantial proportion of patients with HF receive follow-up outside specialised HF clinics and that management within dedicated HF services is associated with more favourable clinical outcomes, including lower CV and all-cause mortality [30]. Although direct comparisons with the present survey are not possible because of substantial methodological differences, both studies highlight the importance of organisational structures capable of ensuring access to specialised expertise and coordinated follow-up. At the same time, they emphasise the heterogeneity that continues to characterise HF management pathways across different healthcare settings.

The growing complexity of HF management further reinforces the importance of dedicated organisational models. Patients with HF are frequently older, affected by multiple CV and non-CV comorbidities, and treated with increasingly complex therapeutic regimens. The implementation and optimisation of GDMT, monitoring of treatment tolerance, management of comorbid conditions, and coordination of follow-up activities require organisational resources that extend beyond the traditional outpatient visit. Within this context, dedicated HF clinics may function not only as clinical services but also as organisational hubs capable of coordinating different phases of the care pathway and facilitating communication among healthcare professionals involved in patient management.

The findings of the present survey indicate that these organisational structures are already widely available within Italian cardiology practice. However, their effectiveness ultimately depends on their ability to interact with the broader healthcare network. The findings of the Regioni 3.0 survey regarding post-discharge follow-up deserve particular attention. More than 90% of respondents reported the availability of structured follow-up pathways, and among these centers the first outpatient reassessment was scheduled within four weeks after discharge in the majority of cases. These data suggest a widespread organisational awareness of the importance of early post-discharge management in patients with HF. Nevertheless, caution is warranted when interpreting these findings. The survey primarily describes the reported availability of organisational pathways rather than their actual implementation in routine clinical practice. As a result, the presence of a structured pathway should not necessarily be interpreted as evidence that all eligible patients undergo timely follow-up according to the planned schedule.

Additional perspective is provided by the BRING-UP HF study [31], which evaluated the implementation of HF management strategies in a broad network of Italian cardiology centers. Compared with the organisational data emerging from the present survey, BRING-UP reported lower rates of early post-discharge reassessment. Rather than representing conflicting findings, these observations should be considered complementary. While the Regioni 3.0 survey explored the organisational availability of follow-up programmes, BRING-UP assessed their practical implementation in routine clinical care. A similar pattern emerged from our own data: although structured post-discharge follow-up pathways were reported by 91.3% of respondents in the Regioni 3.0 survey, only 25.8% of centers with an outpatient HF clinic in the second survey reported reassessing more than 75% of discharged patients within one month of discharge, and 32.0% reassessed fewer than 25%. The presence of a structured pathway should therefore be interpreted as a measure of organisational capacity rather than as evidence of actual timely follow-up delivery. The discrepancy between organisational planning and actual delivery is not unexpected and may be influenced by staffing constraints, outpatient workload, waiting-list pressures, local healthcare organisation, and patient-related factors. Together, these observations suggest that the existence of dedicated pathways represents an essential prerequisite for effective transitional care but does not automatically guarantee their systematic application.

The broader organisational framework described by the 8th ANMCO Census of Italian Cardiology Units provides additional context for interpreting these findings [22]. The Census documented substantial variability in organisational resources across the national territory and highlighted persistent differences in the availability of non-time-dependent care pathways and digital health tools. In this respect, the findings of the present surveys appear consistent with broader national data and suggest that the challenges identified in HF care reflect wider organisational characteristics of the Italian CV healthcare system rather than isolated deficiencies affecting individual centers.

Particularly encouraging are the results of the ANMCO Heart Failure Working group survey regarding discharge planning and outpatient follow-up practices. Medication reconciliation involving the patient and/or caregiver was routinely performed at discharge in 92% of responding centers, while assessment of therapeutic adherence and educational interventions concerning treatment, symptom monitoring, and self-management were routinely incorporated into follow-up activities in approximately 85% of cases. These findings indicate substantial attention to several key components of transitional care and support the growing recognition that successful HF management extends beyond optimisation of pharmacological therapy alone.

The transition from hospital to outpatient care represents a particularly vulnerable period in the natural history of HF. Previous studies have consistently demonstrated that the weeks immediately following hospital discharge are associated with a markedly increased risk of mortality, rehospitalisation, and clinical instability [25,26,27]. During this phase, patients frequently undergo therapeutic adjustments, require reassessment of congestion status and renal function, and may encounter difficulties related to treatment adherence and self-management. Consequently, interventions aimed at improving communication, patient education, medication review, and early clinical reassessment have become central components of contemporary HF programmes [25,26,27,28,29].

Within this context, the high rates of medication reconciliation and adherence assessment reported in the ANMCO Heart Failure Working group survey are particularly relevant. Educational interventions directed at both patients and caregivers may facilitate recognition of warning signs, improve adherence to prescribed therapies, and enhance self-management skills. Although the present surveys were not designed to evaluate clinical outcomes, these organisational practices are consistent with recommendations from contemporary HF guidelines and reflect increasing attention to the quality of transitional care provided by Italian cardiology centers [13,14].

In contrast, the implementation of telemonitoring remains relatively limited. Only approximately one-third of respondents reported the availability of telemonitoring systems for outpatient follow-up. Although the survey did not differentiate among different telemonitoring modalities, this finding suggests that digital health solutions have not yet been fully incorporated into routine HF management. Because the survey item adopted a deliberately broad operational definition (e.g., televisit, structured telephone call, other), the reported 34.1% likely aggregates highly heterogeneous approaches and should be interpreted as an upper-bound estimate of the availability of any form of remote post-discharge follow-up, rather than of structured telemonitoring programmes. Similar observations have emerged from national organisational assessments, indicating that the adoption of telemedicine remains heterogeneous across the Italian healthcare system [22,32].

The significance of this finding extends beyond the simple availability of technology. Telemonitoring may facilitate communication between patients and healthcare providers, support earlier identification of clinical deterioration, improve treatment monitoring, and potentially strengthen connections between hospital-based services and community care. Consequently, the limited diffusion of telemonitoring observed in the present survey may represent one of the principal opportunities for future organisational development. As illustrated in Figure 1 and Figure S1, the challenge appears not only technological but also organisational, requiring dedicated personnel, structured workflows, interoperability between healthcare settings, and sustainable implementation strategies capable of integrating digital tools within existing HF care pathways.

An important observation emerging from both surveys is that many of the organisational components required for effective HF management already exist but are not always integrated into a coordinated network. Dedicated outpatient clinics, structured follow-up programmes, educational interventions, and specialised expertise are widely available; however, stronger interaction between hospital services, primary care physicians, community healthcare providers, and digital health platforms appears necessary to support a more seamless patient pathway. In this regard, the findings summarised in Figure 1, Figure S1 and Figure S2 collectively suggest that the principal challenge for future organisational development is not the creation of new structures, but rather the optimisation and integration of those already in place.

Overall, the surveys support the need for continued efforts to strengthen multidisciplinary resources, improve collaboration between hospital- and community-based services, and promote wider implementation of digital health solutions. Addressing these organisational aspects may contribute to a more homogeneous and coordinated model of HF care across the national healthcare system and may represent an important step towards improving the quality and consistency of care delivered to patients living with HF.

## 5. Limitations

The survey did not distinguish between incident and prevalent patients when quantifying clinical activity volume, nor did it differentiate the activity of dedicated HF outpatient clinics from the overall HF workload of the participating centers. Consequently, the reported patient volumes should be interpreted as a general estimate of HF-related clinical activity rather than a precise measure of specialist outpatient workload.

A further limitation relates to the self-reported nature of the organisational information collected. The survey did not provide standardised operational definitions for several organisational variables, including structured follow-up pathways and functional links with community healthcare services, nor did it include verification of formal institutional documents, codified care pathways, or written operating procedures. Therefore, the findings reflect the respondents’ perception of local organisational structures rather than an objective assessment of the services actually implemented.

In addition, most questionnaires were completed by Directors or Heads of Cardiology Units. Although this approach ensured that respondents were familiar with the organisational characteristics of their institutions, it may also have introduced reporting bias, potentially leading to a more favourable description of existing resources and care pathways.

It was not possible to systematically analyse the organisational characteristics of non-responding centers or to compare them with participating institutions. Consequently, a degree of self-selection bias cannot be excluded, with centers showing greater organisational interest in HF care potentially being more likely to participate. Furthermore, because individual centers were not uniquely identified, the possibility that more than one respondent originated from the same institution cannot be excluded. This may have resulted in the overrepresentation of specific organisational models. Although a post-hoc sensitivity analysis excluding potential duplicate responses yielded essentially unchanged estimates, this approach could not identify respondents from the same institution who provided non-identical answers; therefore, residual institutional duplication cannot be completely ruled out. Accordingly, the unit of analysis was the individual respondent rather than the individual cardiology center, and the findings should be interpreted primarily as descriptive. Furthermore, no patient-level clinical outcomes were collected. Therefore, the relationship between organisational characteristics and clinical outcomes could not be assessed.

The responding centers represent only a proportion of Italian cardiology facilities; therefore, the results cannot be considered fully representative of the national scenario. In this regard, the higher prevalence of dedicated HF outpatient clinics observed in the present survey compared with previous national organisational assessments, including the ANMCO Census, may partially reflect participation bias.

An additional source of potential bias derives from the methodological differences between the two surveys included in the present study. Because individual-level tracking was not performed, it was not possible to determine whether respondents participating in the second survey also contributed to the first survey. Consequently, the two datasets should be considered complementary and descriptive rather than directly comparable. Accordingly, the findings should not be interpreted as a single, fully integrated national assessment of HF care.

Finally, although approximately one-third of respondents reported the availability of telemonitoring systems for patients with HF, the survey did not collect detailed information regarding the type of telemonitoring adopted. Therefore, no distinction could be made between telephone-based follow-up, multiparametric monitoring, or device-based remote monitoring systems, limiting a more detailed interpretation of this finding. Future surveys should adopt standardised definitions distinguishing structured telephone follow-up, televisits, multiparametric remote monitoring, and device-based monitoring. In addition, the surveys did not specifically investigate the availability, organisation, or integration of cardiac rehabilitation programmes within HF care pathways. Although cardiac rehabilitation is recognised as an important component of multidisciplinary HF management and is recommended by current guidelines, no conclusions can be drawn regarding its implementation across participating centres. Future national organisational surveys should include this aspect to provide a more comprehensive assessment of HF care networks.

## 6. Future Perspectives

Although descriptive and cross-sectional in nature, the present study provides a contemporary overview of the organisational landscape of HF care in Italy and may serve as a foundation for future national assessments. In an evolving healthcare environment characterised by population ageing, increasing multimorbidity, and the progressive adoption of digital health technologies, the systematic evaluation of organisational indicators is becoming increasingly important alongside the monitoring of clinical outcomes.

Future initiatives should focus on the development of standardised organisational metrics capable of assessing the availability of multidisciplinary resources, the degree of integration between hospital and community services, and the implementation of digital health solutions. Repeated national surveys using harmonised methodologies could allow longitudinal evaluation of organisational changes over time and facilitate comparisons across different healthcare settings. In this context, ANMCO may contribute to promoting a shared framework for the periodic assessment of HF care organisation at a national level.

## 7. Conclusions

The findings of these two complementary exploratory surveys provide a descriptive overview of the current organisation of HF care in Italy. Dedicated HF outpatient clinics and structured post-discharge follow-up pathways are widely available, indicating that several key components of contemporary HF management have been successfully implemented within Italian cardiology practice. Nevertheless, the organisational availability of these services should be distinguished from their actual clinical implementation, as the presence of structured pathways does not guarantee that all eligible patients receive timely follow-up.

However, important organisational gaps remain. Dedicated non-physician personnel, particularly specialised HF nurses, are not uniformly available, formal integration with community healthcare services remains limited, and telemonitoring systems are implemented in only a minority of centers. Geographical differences appear to be driven primarily by variability in organisational resources and network connectivity rather than by differences in the availability of specialised HF services.

These findings support the need for continued efforts to strengthen multidisciplinary care, improve collaboration between hospital- and community-based healthcare providers, and promote the wider adoption of digital health tools. Optimising the integration of existing organisational resources represents a realistic and potentially high-impact strategy to improve the quality and consistency of HF care across the Italian healthcare system. It should be acknowledged, however, that the present study did not include patient-level data, clinical outcomes, readmission rates after the implementation of care pathways, or objective verification of written protocols; therefore, the potential benefit of specific organisational models on clinical outcomes should be regarded as a plausible implication, supported by the existing literature on multidisciplinary HF care, and warrants confirmation in prospective, outcome-based studies.

## Figures and Tables

**Table 1:** Characteristics and organisational resources of participating centers (*n* = 196).

Variable	*n* (%)
Professional qualification	
Director of Complex Cardiology Unit	163 (84.0)
Medical Doctor/Manager	24 (12.4)
Head of Simple Unit	7 (3.6)
Type of center	
Cardiology with beds and ICCU	149 (76.4)
Cardiology with beds without ICCU	28 (14.4)
Other organisational setting	13 (6.7)
Cardiology without beds	5 (2.6)
Organisational resources	
Dedicated HF outpatient clinic	168 (85.7)
Dedicated non-physician staff	83 (42.6)
Dedicated HF nurses	75 (38.5)
Other dedicated healthcare professionals	8 (4.1)
Day Hospital/Day Service or MAC for HF	104 (53.1)
Functional links with GPs/community services or dedicated networks	93 (47.7)
Monthly HF patient volume	
<50 patients/month	77 (39.3)
50–100 patients/month	99 (50.5)
>100 patients/month	20 (10.2)

The footer of the tables uses a light grey background to visually distinguish explanatory notes, methodolog-ical clarifications, and abbreviations from the main data rows, thereby improving readability and prevent-ing these elements from being interpreted as part of the reported results. This is particularly useful for notes specifying denominators or conditional responses.

**Table 2:** Geographical stratification of organisational characteristics of heart failure care in Italy.

Variable	North (*n* = 98)	Center (*n* = 40)	South & Islands (*n* = 58)	*p*-Value
Dedicated HF outpatient clinic	88/98 (89.8%)	35/40 (87.5%)	45/58 (77.6%)	0.102
Dedicated non-physician staff	49/97 (50.5%)	10/40 (25.0%)	24/58 (41.4%)	0.022
Dedicated HF nurses	44/97 (45.4%)	10/40 (25.0%)	21/58 (36.2%)	0.077
Day Hospital/Day Service or MAC for HF	63/98 (64.3%)	16/40 (40.0%)	25/58 (43.1%)	0.007
Functional links with GPs, community services, or dedicated networks	60/97 (61.9%)	11/40 (27.5%)	22/58 (37.9%)	<0.001
Structured post-discharge follow-up	93/98 (94.9%)	36/40 (90.0%)	50/58 (86.2%)	0.166
First follow-up visit ≤ 4 weeks *	78/93 (83.9%)	33/36 (91.7%)	48/53 (90.6%) 47/50 (94.0%)	0.155

* Among centers reporting a structured post-discharge follow-up pathway. Abbreviations: HF, heart failure; GP, general practitioner; MAC, complex outpatient activity unit. The background color in the table footer is used solely to visually distinguish the explanatory notes and abbreviations from the main body of the table and to improve readability. It does not convey any additional meaning or represent a separate data category. The same formatting has been applied consistently to the corresponding table footers throughout the manuscript.

**Table 3:** Stratification of organisational characteristics according to center type (*n* = 196).

Variable	Cardiology with Beds and ICCU (*n* = 149)	Cardiology with Beds Without ICCU (*n* = 28)	Cardiology Without Beds (*n* = 5)	Other Settings (*n* = 13)	*p*-Value
Dedicated HF outpatient clinic	136/149 (91.3%)	21/28 (75.0%)	3/5 (60.0%)	7/13 (53.8%)	<0.001
Dedicated non-physician staff	64/149 (43.0%)	10/27 (37.0%)	3/5 (60.0%)	5/13 (38.5%)	0.788
Dedicated HF nurses	61/149 (40.9%)	10/27 (37.0%)	2/5 (40.0%)	1/13 (7.7%)	0.131
Day Hospital/Day Service or MAC for HF	85/149 (57.0%)	11/28 (39.3%)	2/5 (40.0%)	5/13 (38.5%)	0.209
Functional links with GPs, community services, or dedicated networks	71/149 (47.7%)	11/27 (40.7%)	2/5 (40.0%)	8/13 (61.5%)	0.651
Structured post-discharge follow-up	144/149 (96.6%)	22/28 (78.6%)	2/5 (40.0%)	10/13 (76.9%)	<0.001
First follow-up visit ≤ 4 weeks *	133/144 (92.4%)	16/22 (72.7%)	2/2 (100.0%)	6/10 (60.0%)	0.002

* Among centers reporting a structured post-discharge follow-up pathway. Abbreviations: HF, heart failure; ICCU, intensive cardiac care unit; GP, general practitioner; MAC, complex outpatient activity unit. The background color in the table footer is used solely to visually distinguish the explanatory notes and abbreviations from the main body of the table and to improve readability. It does not convey any additional meaning or represent a separate data category.

**Table 4:** Referral pathways and post-discharge follow-up organisation (*n* = 196).

Variable	*n* (%)
Referral pathways *	
Emergency department	136 (69.4)
GP outpatient clinic	98 (50.0)
Internal medicine/geriatrics	97 (49.5)
Territorial cardiology clinic	84 (42.9)
Other sources	73 (37.2)
Structured follow-up	
Yes	179 (91.3)
No	17 (8.7)
First post-discharge visit †	
≤4 weeks	158 (88.3)
>4 weeks	21 (11.7)
Timing of reassessment after discharge	
1 week	15 (7.8)
1 month	151 (78.2)
6 months	17 (8.8)
12 months	1 (0.5)
Referred elsewhere	9 (4.7)
Follow-up destination	
Hospital HF clinic	166 (85.6)
Community cardiologist	11 (5.7)
GP	9 (4.6)
Private cardiologist	5 (2.6)
Private/intramural outpatient clinic	3 (1.5)
Average annual reassessments	
0	3 (1.5)
1	10 (5.2)
2	63 (32.5)
3	96 (49.5)
≥4	22 (11.3)

* Multiple responses allowed. Percentages are not mutually exclusive. † Among the 179 respondents reporting a structured post-discharge follow-up pathway (item 10 = Yes). Five respondents who answered this item despite reporting no structured pathway were excluded. The background color in the table footer is used solely to visually distinguish the explanatory notes and abbreviations from the main table content and to improve readability. It does not convey any additional meaning or indicate a separate data category.

**Table 5:** ANMCO Heart Failure Working Group survey: discharge planning and outpatient follow-up practices (*n* = 135).

Item	Main Finding
Routine medication reconciliation involving the patient and/or caregiver at discharge	91.9%
Availability of a dedicated outpatient HF clinic	86.7%
Availability of a dedicated HF nurse	43.7%
Proportion of discharged patients reassessed within 1 month †	<25%: 32.0%; 25–50%: 20.3%; 50–75%: 21.9%; >75%: 25.8%
Systematic assessment of therapeutic adherence and/or educational intervention†	Both: 46.6%; Adherence only: 23.7%; Education only: 16.8%; Neither: 13.0%
Availability of a telemonitoring system in the post-discharge vulnerable period	34.1%

The background color in the table footer is used solely to visually distinguish the explanatory notes and ab-breviations from the main table content and to improve readability. It does not convey any additional meaning or indicate a separate data category. † is used to identify the specific footnote applicable to the corresponding table entry and to distinguish it from the other footnote(s). In particular, it indicates that the reported percentages were calculated among the eligible respondents after excluding responses that were not applicable.

## Data Availability

The data supporting the findings of this study are available from the corresponding author upon reasonable request. The data are not publicly available because they consist of anonymised survey responses collected for organisational purposes.

## Supplementary Materials

The following supporting information can be downloaded at: https://www.mdpi.com/article/10.3390/jcm15218551/s1, Table S1, Regioni 3.0 survey questionnaire on the organisation of outpatient heart failure care, distributed to Directors of Complex Cardiology Units, Heads of HF Centers, and other cardiologists with organisational responsibilities across the ANMCO Regional Sections; Table S2, ANMCO Heart Failure Working Group survey questionnaire on discharge planning and outpatient follow-up management in patients hospitalised for acute heart failure, administered during the Heart Failure Centers Convention (Milan, 25–26 October 2024); Figure S1: Geographical distribution of Day Hospital/Day Service/complex outpatient activity unit (MAC) facilities dedicated to heart failure patients and of functional links with general practitioners, community healthcare services, and dedicated heart failure networks, according to macro-area (Northern, Central, and Southern Italy); Figure S2: Referral pathways to specialised heart failure services and organisation of post-discharge outpatient follow-up.
