# Supplementary material for: Chronic Heart Failure Care Network in Italy: Results from the Regioni 3.0 and ANMCO Heart Failure Working Group Surveys
Source: J Clin Med. 2026 Nov 3;15(21):8551. doi: 10.3390/jcm15218551 (PMC13764513; doi:10.3390/jcm15218551)
Supplement: Supplementary file 1 [file jcm-15-08551-s001.zip › jcm-4406474-supplementary.pdf]

Supplementary Material

Chronic Heart Failure Care Network in Italy: Results from the Regioni 3.0 and ANMCO Heart Failure Working Group Surveys

Both surveys were originally administered in Italian; the questionnaires are reported below in English translation.

Supplementary Table S1. ANMCO Regioni 3.0 survey: "Chronic Heart Failure Network between Hospital and Community". Full questionnaire

The survey consisted of 14 items and was distributed between November 2023 and September 2024 to 563 ANMCO members with organisational responsibilities in heart failure (HF) care (Directors of Complex Cardiology Units, Heads of HF Centers, and other cardiologists with direct organisational responsibilities), through the ANMCO Regional Sections. A total of 196 responses were collected (response rate 34.8%). Unless otherwise specified, items allowed a single response; item 9 allowed multiple responses.

Item	Response options / type
1. Indicate the Region where you work.	**Single response**<br>List of the 20 Italian Regions / Autonomous Provinces
2. Indicate your professional role.	**Single response**<br>– Director of Complex Cardiology Unit<br>– Head of Simple Unit<br>– Medical Doctor / Manager
3. Indicate the type of center where you work.	**Single response**<br>– Cardiology with inpatient beds and ICCU<br>– Cardiology with inpatient beds without ICCU<br>– Cardiology without inpatient beds<br>– Other
4. Is there a dedicated chronic heart failure (CHF) outpatient clinic at the center where you work?	**Single response**<br>– Yes<br>– No
5. Are there non-physician professionals exclusively dedicated to heart failure at your center?	**Single response**<br>– No<br>– Nurses<br>– Other healthcare professionals
6. Is there a Day Hospital and/or Day Service or complex outpatient activity unit (MAC) for CHF patients at your center?	**Single response**<br>– Yes<br>– No
7. Are there functional links between your CHF outpatient clinic and general practitioners (GPs) or community healthcare services, or a dedicated network?	**Single response**<br>– Yes<br>– No
8. How many CHF patients are managed at your center per month?	**Single response**<br>– <50<br>– 50–100<br>– >100
9. How does the patient with heart failure come to your attention?	**Multiple responses allowed**<br>– From the emergency department<br>– From Internal Medicine/Geriatrics<br>– From the GP outpatient clinic<br>– From territorial/community outpatient

Item	Response options / type
	clinics<br>– Other<br>*Percentages are not mutually exclusive; denominator = total number of respondents.*
10. Is there a structured follow-up pathway for the management of the CHF patient at discharge?	**Single response**<br>– Yes<br>– No
11. If you answered Yes to the previous question, is the first post-discharge visit scheduled within 4 weeks?	**Single response**<br>– Yes<br>– No<br>*Answered only by respondents reporting a structured follow-up pathway (item 10 = Yes).*
12. When do you reassess the CHF patient after hospitalisation?	**Single response**<br>– At 1 week<br>– At 1 month<br>– At 6 months<br>– At 12 months<br>– Referral to another center and/or to the GP and/or to a trusted private cardiologist
13. How is post-discharge follow-up of the CHF patient managed and which are the reference figures in your territory?	**Single response**<br>– I refer the patient to the hospital HF outpatient clinic<br>– I refer the patient to a community cardiologist<br>– I refer the patient to the GP<br>– I refer the patient to a trusted private cardiologist<br>– I reassess the patient at my private or intramural outpatient clinic
14. On average, how many times per year is the CHF patient reassessed at your center after hospitalisation?	**Single response**<br>– 0<br>– 1<br>– 2<br>– 3<br>– ≥4

Abbreviations: CHF, chronic heart failure; HF, heart failure; ICCU, intensive cardiac care unit; GP, general practitioner; MAC, complex outpatient activity unit.

Supplementary Table S2. ANMCO Heart Failure Working Group survey: "Management of the patient at discharge and during follow-up after hospitalisation for Acute Heart Failure" . Full questionnaire

The survey consisted of six single-response, closed-ended items and was administered during the Heart Failure Centers Convention held in Milan on 25–26 October 2024 and distributed to ANMCO members. A total of 135 responses were collected. For items 4 and 5, respondents without a dedicated outpatient HF clinic could select a "not applicable" option; these responses were excluded from the denominator when calculating item-specific percentages.

Item	Response options / type

Item	Response options / type
1. At discharge, is medication reconciliation involving the patient and/or caregiver routinely performed by a physician and/or nurse?	**Single response**<br>– Yes<br>– No
2. Is there an outpatient clinic for heart failure management at your center?	**Single response**<br>– Yes<br>– No
3. Is there a nurse dedicated to heart failure management at your center?	**Single response**<br>– Yes<br>– No
4. What proportion of patients hospitalised for heart failure are you able to reassess in the outpatient setting within one month of discharge?	**Single response**<br>– <25%<br>– 25–50%<br>– 50–75%<br>– >75%<br>– Not applicable (e.g. no outpatient clinic available)<br>*“Not applicable” responses (n = 7) were excluded from the denominator (n = 128).*
5. In your outpatient setting, is patient adherence systematically assessed and is an educational intervention on therapy and on self-monitoring of symptoms and clinical parameters performed?	**Single response**<br>– Yes, both<br>– Yes, I systematically assess adherence to recommendations<br>– Yes, I systematically perform an educational intervention on therapy and self-monitoring<br>– No<br>– Not applicable (e.g. no outpatient clinic available)<br>*“Not applicable” responses (n = 4) were excluded from the denominator (n = 131).*
6. Is there a patient telemonitoring system at your center (e.g. televisit, structured phone call, other) during the post-discharge vulnerable period?	**Single response**<br>– Yes<br>– No

Abbreviations: HF, heart failure.

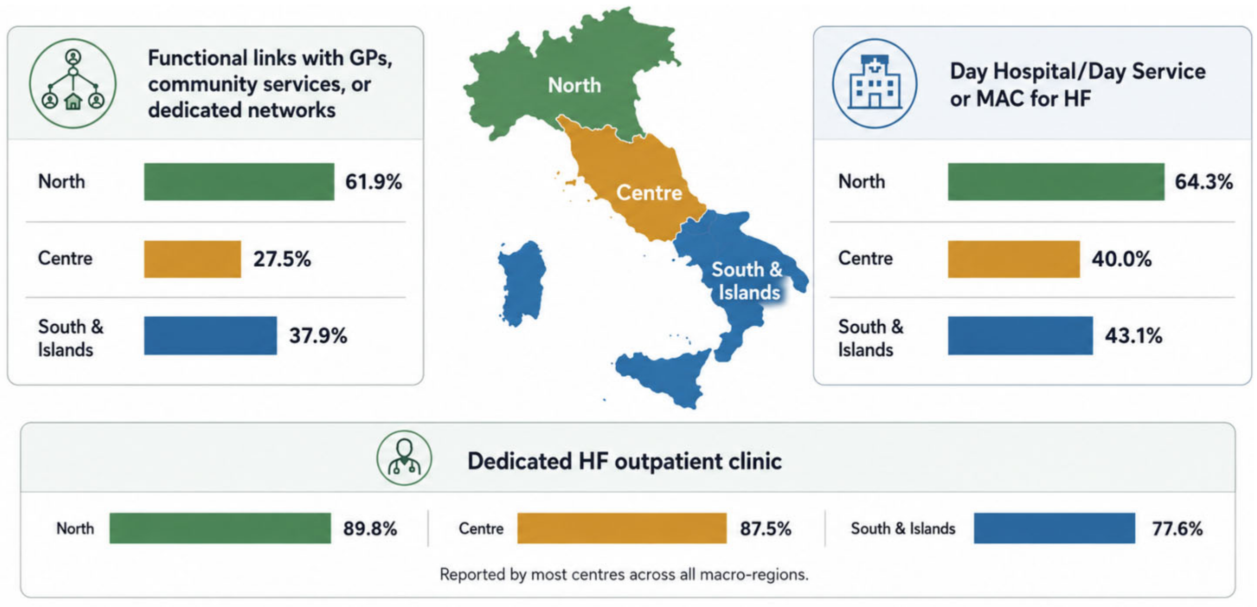

Supplementary Figure S1. Geographical Variations in the Organisation of Heart Failure Care Across Italy.

The figure illustrates regional differences in selected organisational characteristics of heart failure (HF) care across Italy. Northern regions reported higher availability of functional links with general practitioners (GPs), community healthcare services, or dedicated HF networks, as well as greater availability of Day Hospital/Day Service or complex outpatient activity units (MAC) dedicated to HF management. In contrast, lower rates were observed in Central and Southern regions. The availability of dedicated HF outpatient clinics remained consistently high across all macro-regions, indicating broad diffusion of specialised HF services nationwide despite differences in organisational integration and intermediate-care resources.

Abbreviations: HF: Heart Failure; GP: General Practitioner; MAC: Complex Outpatient Activity Unit

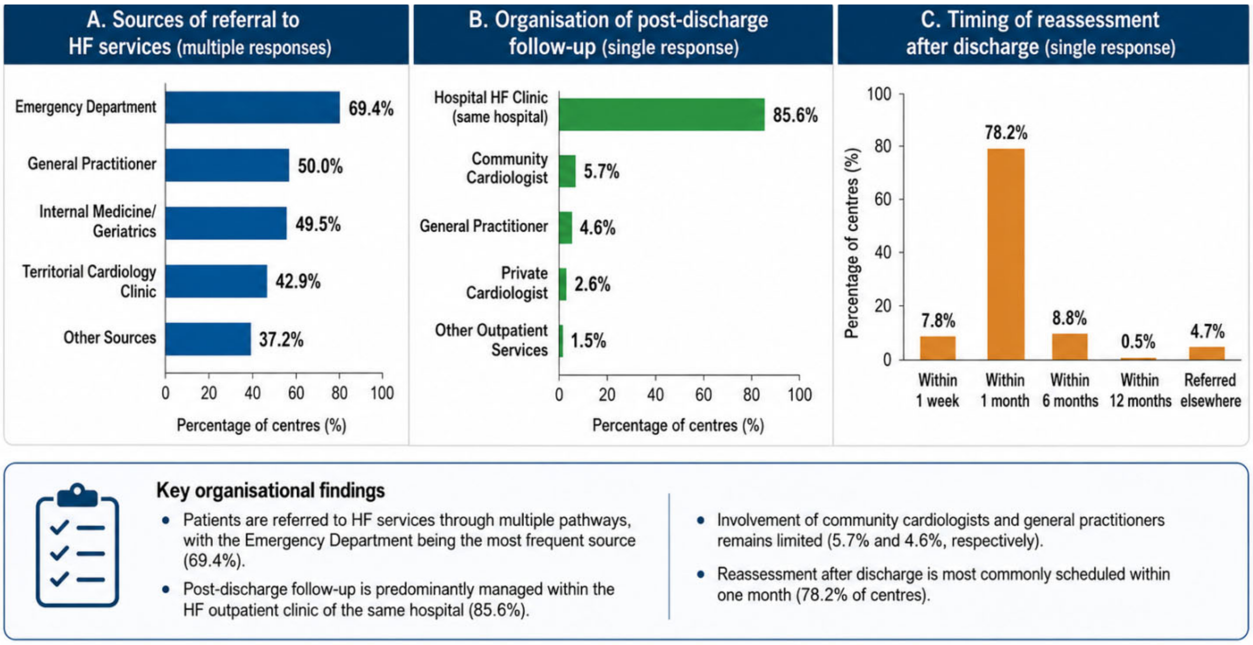

Supplementary Figure S2. Referral Pathways and Organisation of Post-Discharge Follow-Up in Heart Failure Care.

The figure summarises referral pathways to HF services and the organisation of post-discharge follow-up reported in the Regioni 3.0 survey. Patients were referred to HF services through multiple sources, including emergency departments, general practitioners, internal medicine or geriatrics departments, territorial cardiology clinics, and other healthcare settings. Follow-up after hospital discharge was predominantly performed within the HF outpatient clinic of the same institution, whereas referral to community cardiologists and general practitioners was reported less frequently. Reassessment after discharge was most commonly scheduled within one month. The figure provides an overview of the organisational characteristics of patient referral and follow-up pathways across participating Italian cardiology center

HF: Heart Failure, GP : General Practitioner
